# Final-Size Solutions for SIRI Models with Vaccination

**DOI:** 10.1007/s11538-026-01610-w

**Published:** 2026-03-24

**Authors:** Maria A. Gutierrez, Julia R. Gog

**Affiliations:** 1 Odum School of Ecology, University of Georgia, Athens, USA; 2 Department of Applied Mathematics and Theoretical Physics, University of Cambridge, Cambridge, UK

**Keywords:** Compartmental models, Epidemic model, Final-size solution, Partial immunity, Reinfection threshold, Vaccination

## Abstract

In the classic SIR model, infection gives full immunity against any possible reinfection. However, for many important epidemiological situations, immunity is only partial and reinfection is possible. Though these models are mathematically more complex, we are able to find expressions for the epidemic final size. We also generalise these expressions to include vaccination, with a fraction of the population vaccinated before the epidemic, where vaccinees are less susceptible to primary infections than unvaccinated hosts.

Partial immunity can be interpreted at the population level as providing either full or no protection to each host, in some proportion (all-or-none immunity). In this scenario, we give analytical expressions (mathematically similar to the SIR final-size) for the cumulative primary infections and the cumulative reinfections in unvaccinated and vaccinated hosts. Alternatively, partial immunity can be interpreted as providing homogeneous imperfect protection to each host (leaky immunity). For this other scenario, we again obtain an implicit equation for the final epidemic size. We break down, in terms of the final size, the number of infections in hosts with or without prior immunity (vaccine- or infection- induced), as well as the number of primary infections and reinfections. Under the leaky immunity assumption, we find a form of reinfection threshold. If the relative host susceptibility to reinfection is above this threshold (which is the inverse of the pathogen’s basic reproduction number), transmission rates are high enough to support an endemic disease. Below the reinfection threshold, epidemics are transient. In the all-or-none model, epidemics are always transient.

## Introduction

The classic SIR epidemic model, first analysed in Ross and Hudson (1917), includes the simplifying assumption that infection confers total immunity (Keeling and Rohani 2007). However, many pathogens do not provide complete immunity: reinfection is possible (Finlay and McFadden 2006). Moreover, there is some evidence of sequential infections with the same strain in both Influenza A (Memoli et al. 2020) and SARS-CoV-2 (Tillett et al. 2021), although waning immunity may play an important role here. The Omicron SARS-CoV-2 variant provides especially low protection against reinfection (Powell et al. 2023). Reinfections can be key in shaping the epidemic dynamics of some pathogens, such as SARS-CoV-2 (Kissler et al. 2020). As a rule of thumb, epidemics will be prolonged if reinfection is possible. Furthermore, some pathogens become endemic due to the lack or loss of protection against reinfection (Gomes et al. 2004).

Deterministic compartmental models are widely used in epidemiology to study the spread of infectious diseases (Keeling and Rohani 2007). A key advantage of these simple ODE models is that they allow direct access to the “final size” of an epidemic: the cumulative number of infections in the host population. Final-size expressions allow more complex phenomena to be explored; for example, the evolution of a disease across multiple seasons (Boni et al. 2004; Andreasen 2003) or the evolutionary pressure acting on a pathogen over a single epidemic wave (Gutierrez and Gog 2023). An expression for the final size of a classical SIR epidemic can be derived in terms of the Lambert W function (Lehtonen 2016), and this final-size solution can be generalised in various ways (Ma and Earn 2006; Gog and Hollingsworth 2021; Gutierrez and Gog 2023).

A common relaxation of the full immunity assumption is that infection induces full but temporary immunity (Keeling and Rohani 2007). The biological mechanism here could be loss of immunity, or the antigenic evolution of the circulating pathogen (Andreasen and Gog 2020; Pease 1987). These SIRS-type models do not usually have final-size solutions because the epidemic does not burn out. Instead, a positive endemic equilibrium is possible because waning of immunity replenishes the pool of susceptible individuals, offering a dynamic balance to susceptible depletion through infection (Keeling and Rohani 2007).

In this paper, we instead consider the alternative assumption of lifelong but imperfect immunity: infection offers some protection against further infections, but reinfection is still possible. These “SIRI”-type epidemic dynamics often show a transient epidemic. We obtain analytic final-size expressions for the total infections over the course of the epidemic, split into primary infections and reinfections. This is also extended to vaccination, and then the final size is further disaggregated by host vaccination status. We consider two interpretations of the “partial” nature of immunity: “all-or-none” and “leaky” immunity (Gomes et al. 2014).

## General Modelling Assumptions

We use deterministic compartmental models to find the cumulative number of infections during an epidemic wave. We assume a single strain of an infectious disease and a well-mixed population of constant size, with no births or deaths. We choose time units to make the recovery rate one, which we assume is the same for all hosts (regardless of immune status). We consider an epidemic that is unmitigated, apart from the use of vaccines. We assume that a proportion *c* of the population is vaccinated before the epidemic (Table 1). We consider only vaccines that provide partial but lifelong immunity (explained below), so vaccinated individuals may still become infected. Up to this point, these assumptions follow those in Gutierrez and Gog (2023).

We assume that both infection- and vaccine-induced immunity reduce host susceptibility but not necessarily by the same amount, similarly to the “partial immune protection” model of Gomes et al. (2004). We use $\theta _S$ to denote the relative susceptibility of vaccinees: $\theta _S=1$ means no protection against infection. Analogously, we use $\phi _S$ for the relative susceptibility of recovered hosts ($\phi _S=1$ means no protection against reinfection). Thus, unless $\phi _S=0$, reinfections are possible, in contrast to Gutierrez and Gog (2023) where recovered individuals are fully protected. We assume that prior immunity does not change host infectiousness.

**Table 1:** Parameters and output functions

item	description
$\theta _S \in [0,1]$	relative susceptibility (to a first infection) of vaccinees
$\phi _S \in [0,1]$	relative susceptibility of recovered hosts, analogous to $\theta _S$
$c \in [0,1]$	vaccination coverage of the population
$$R_0 $$	initial basic reproduction number, assumed $$R_0>1$$
$R_e(c)$	$R_e=R_0(1-c(1-\theta _S))$, initial effective reproduction number, assumed $R_e>1$
$$c_h $$	vaccination coverage threshold for $$R_e(c_h)=1$$, $$c_h=(1-R_0^{-1})/(1-\theta _S)$$
*r*(*c*)	$(R_e-\phi _S)/(1-\phi _S)$, similar to $R_e$, but including reinfections for $\phi _S>0$
$C_U(c)$	total primary infections in unvaccinated hosts
$C_V(c)$	total primary infections in vaccinated hosts
$C^\dagger _U(c)$	total reinfections in unvaccinated hosts
$C^\dagger _V(c)$	total reinfections in vaccinated hosts

## Model with All-or-None Immunity

### Model Description

For the all-or-none model assumption, the relative susceptibility factors ($\theta _S$ and $\phi _S$) are interpreted as all-or-none immunity (also termed “polarised” or “all-or-nothing” immunity) (Gomes et al. 2014; Gog and Swinton 2002; Halloran et al. 1999). With all-or-none vaccine-induced immunity, a proportion $1-\theta _S$ of the vaccinated individuals become fully protected against the disease. The remaining $\theta _S$ vaccinated individuals are still fully susceptible to the disease. Similarly, after infection, an individual becomes fully immune with probability $1-\phi _S$ or returns to the susceptible compartment with probability $\phi _S$. Section 4 instead uses leaky immunity, as in Gomes et al. (2004) and other “SIRI”-type (Susceptible-Infected-Recovered-Infected) models (e.g., Moreira and Yuquan 1997).

We refer to the vaccinated and unvaccinated with the subscripts *U* and *V*. We use the superscript $\dagger$ for individuals who have been infected before. The compartments of the model are thus $S_U, S^\dagger _U, S_V, S^\dagger _V$, the proportions of the population fully susceptible to the disease, and $I_U, I_V$, the infected proportions. $R_0$ is the basic reproduction number and *c* is the vaccinated proportion of the population. The ODE model (Fig. 1) is

1 $$\begin{aligned} \dot{S}_*&= -S_* \lambda (t) \end{aligned}$$

2 $$\begin{aligned} \dot{S}_*^\dagger&= - S_*^\dagger \lambda (t) + \phi _S I_* \end{aligned}$$

3 $$\begin{aligned} \dot{I}_*&= (S_*+S_*^\dagger ) \lambda (t) -I_* \end{aligned}$$

for each of $*=U,V$ and $\lambda (t) = R_0(I_U + I_V)$ is the force of infection and the dots denote differentiation with respect to time. We assume that all unvaccinated individuals are initially susceptible, except for a few infections that initiate the outbreak. Similarly, we assume that a proportion $\theta _S$ of those vaccinated is fully susceptible from the start of the outbreak (as in the all-or-none models of Gutierrez and Gog (2023)). Therefore, the initial conditions are

4 $$\begin{aligned} S_U(0)&=1-c &  S_V(0)=c \theta _S &  S^\dagger _U(0) = S^\dagger _V(0) =0&\nonumber \\&I_U(0) = \epsilon _U \ll 1 &  I_V(0) =\epsilon _V \ll 1 \end{aligned}$$

where $\epsilon _U, \epsilon _V \ge 0$ represent the infected hosts that initiate the outbreak (at least one of $\epsilon _U, \epsilon _V$ is positive). The final-size calculation (Section 3.2) later sets these infinitesimal proportions of the population to zero, as in the standard approach to final-size calculations (Keeling and Rohani 2007).

**Fig. 2:**
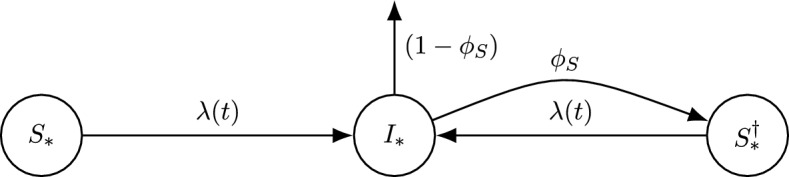
Schematic representation of the SIRI all-or-none model. The movement of individuals between compartments is analogous for the unvaccinated ($*=U$) and vaccinated ($*=V$). Flow rates between compartments are given per capita (and with the average infectious period as time unit). The arrow with rate $1-\phi _S$ out of $I_*$ represents those hosts who become fully immune upon recovery from infection

### Final-Size Calculation

The vaccinated and unvaccinated populations are subject to the same epidemic dynamics (1)-(3). Thus, they stay locked in proportion to each other (as per Appendix A.1 of Gutierrez and Gog (2023)), according to the initial conditions (4):

5 $$\begin{aligned} (S_V, S^\dagger _V, I_V)= \frac{c \theta _S}{1-c} (S_U, S^\dagger _U, I_U ). \end{aligned}$$

provided that $(1-c)\epsilon _V=c\theta _S\epsilon _U$ (which we assume to be true since $\epsilon _U, \epsilon _V \ll 1$). We define new variables $(S,S^\dagger , I)=(1-c)^{-1} (S_U, S^\dagger _U, I_U)$ and use (5) to obtain

6 $$\begin{aligned} \dot{S}&= -S \lambda&S(0)=1 \end{aligned}$$

7 $$\begin{aligned} \dot{S}^\dagger&= -S^\dagger \lambda + \phi _S R_e^{-1} \lambda&S^\dagger (0)=0\end{aligned}$$

8 $$\begin{aligned} \dot{I}&= R_e (S+S^\dagger ) I -I&0< I(0)=\epsilon \ll 1 \end{aligned}$$

where $\lambda =R_e I$ and $R_e=R_0 [1-c(1-\theta _S )]$ is the initial effective reproduction number. For $\theta _S<1$, increasing the vaccinatoon coverage *c* lowers $R_e$. If the vaccination coverage is above its critical value $c_h= (1-R_0^{-1})/(1-\theta _S ),$ the effective reproduction number is less than one ($R_e<1$). Therefore, herd immunity prevents an outbreak for a vaccination coverage above this threshold ($c>c_h$): the prevalence decreases exponentially. If, instead, the vaccination coverage is below the threshold ($c<c_h$), the epidemic grows. To obtain the final size of the epidemic, we aim to express *I* in terms of *S* only (we use mathematical manipulations similar to those involved in the traditional derivation of the SIR final-size result, as the models here are not amenable to analysis with some other final-size approaches (Miller 2012)). Using the chain rule,

9 $$\begin{aligned} \frac{dS^\dagger }{dS} =\frac{\dot{S}^\dagger }{\dot{S}}&= -\frac{\phi _S}{R_e S}+\frac{S^\dagger }{S}. \end{aligned}$$

Multiplying both sides of (9) by 1/*S* we see that $\frac{d}{dS} (S^\dagger /S) = -\phi _S / R_e S^2$, which we integrate using the initial conditions $S^\dagger =0$ and $S=1$ at $t=0$. Hence, $S^\dagger /S = {\phi _S}(1/S-1)/R_e$ and thus

10 $$\begin{aligned} S^\dagger&=\phi _S R_e^{-1}(1-S). \end{aligned}$$

As expected, $S^\dagger$ in (10) is a decreasing function of *S*, because *S* (a proxy for the proportion of hosts who have never been infected) continuously decreases throughout the epidemic while $S^\dagger$ (recovered hosts) increases. Using (10), the ODE for *I*(*t*) in (8) becomes

11 $$\begin{aligned} \dot{I} = (R_e -\phi _S)I S -(1-\phi _S)I. \end{aligned}$$

By comparison with the standard SIR equations, the ODE (11) suggests that ${r=(R_e-\phi _S)/({1-\phi _S})}$ may determine the epidemic dynamics here in a similar way to how the basic reproduction number $R_0=\beta /\gamma$ affects the SIR dynamics: Section 3.3 discusses this analogy further. To calculate the final epidemic size, we use (11) and (6):

12 $$\begin{aligned} \frac{dI}{dS}&= \frac{\dot{I}}{\dot{S}} = -\left( 1-\frac{\phi _S}{R_e} \right) + \frac{1-\phi _S}{R_e S}. \end{aligned}$$

Using the initial conditions $I = 0$ and $S=1$ at $t=0$, we obtain

13 $$\begin{aligned} I = \left( 1-\frac{\phi _S}{R_e} \right) (1-S)+\frac{1-\phi _S}{R_e}\log {S}. \end{aligned}$$

To find the final size $S^{\infty }:= \lim _{t\rightarrow \infty } S(t) <1$, we take $t \rightarrow \infty$, and set $\lim _{t\rightarrow \infty } I(t)=0$. Thus,

14 $$\begin{aligned} S^{\infty }&= 1-\frac{1-\phi _S}{R_e-\phi _S}\log {\frac{1}{S^{\infty }}} = -\frac{1}{r} W[-re^{-r}] \end{aligned}$$

where $r=(R_e-\phi _S)/(1-\phi _S)$, as above, and *W* is the (principal branch of the) Lambert W function (Lehtonen 2016): obeying $W(x)e^{W(x)}$ and $W(x) \in [-1,0]$ for $x \in [e^{-1},0]$. The cumulative number of primary infections in the unvaccinated $C_U=(1-c-\lim _{t \rightarrow \infty } S_U)$. Since $C_U$ counts infections across the full epidemic, it does not depend on the time *t*. By definition $S_U=(1-c)S$, hence $C_U=(1-c)(1-S^{\infty })=(1-c) \left( 1+ W[-re^{-r}]/r \right)$. The cumulative reinfections in the unvaccinated are

15 $$\begin{aligned} C^\dagger _U=(1-c) \int _0^{\infty } S^\dagger \lambda dt = (1-c) \int _{S^{\infty }}^1 \frac{S^\dagger }{S}dS \end{aligned}$$

which we integrate using (10) and (14), so that $C^\dagger _U{=}(1{-}c) \frac{\phi _S}{R_e}(r{-}1) \left( 1{+} W[-re^{{-}r}]/r \right)$.

### Final-Size Results

For vaccination coverages below the herd-immunity threshold ($c<c_h$), the calculations above show that the cumulative primary infections in unvaccinated individuals are

16 $$\begin{aligned} C_U =(1-c)\left( 1+\frac{1}{r} W\left[ -r e^{-r}\right] \right) \text { with }r = \frac{R_e(c)-\phi _S}{1-\phi _S}>1 \end{aligned}$$

where *W* is the Lambert W function (Lehtonen 2016) and $0 \le \phi _S<1$. The proportions (5) imply that the cumulative primary infections in vaccinated individuals are $C_V=c\theta _SC_U/(1-c)$. If past infections do not protect at all against future infections ($\phi _S=1$), the final-size calculation (16) is not valid (because $r=\infty$). Instead, with $\phi _S=1$, we have an SIS model (Keeling and Rohani 2007). For $r \in (1, \infty )$, the factor ${f(r)=(1+W\left[ -r e^{-r}\right] /r)}$ in (16) is the final size of a standard SIR epidemic with initial reproduction number *r* (Lehtonen 2016). Therefore, even though $R_e$ is the actual initial effective reproduction number, *r* plays a more important role here with reinfections ($\phi _S > 0$), as the ODE (11) suggests. Unfortunately, we do not have a clear biological interpretation of *r*. Without reinfections ($\phi _S=0$), *r* is just $R_e$.

As the probability of remaining susceptible upon recovery ($\phi _S$) increases from zero, transmission increases: $r>R_e$ monotonically increases with increasing $\phi _S$. Therefore, with reinfections ($\phi _S>0$) there are more primary infections $C_U, C_V$ than without reinfections ($\phi _S=0$). Both $C_U$ and $C_V$ monotonically increase with increasing $\phi _S$, because a higher $\phi _S$ requires more infections in the population to build herd immunity (which overturns the epidemic).

We have also shown above that the cumulative reinfections in unvaccinated hosts are

17 $$\begin{aligned} C^\dagger _U= \frac{\phi _S}{R_e}(r-1) (1-c)\left( 1+\frac{1}{r} W\left[ -r e^{-r}\right] \right) \end{aligned}$$

for $c<c_h$. Using (5), $C^\dagger _V=c\theta _S C^\dagger _U/(1-c)$ are the reinfections in vaccinated individuals. Unsurprisingly both $C^\dagger _U$ and $C^\dagger _V$ are increasing functions of $\phi _S$ (lower protection against reinfection means there are more reinfections).

The final-size expressions (16) and (17) show that the ratio of reinfections to primary infections is

18 $$\begin{aligned} \frac{C_*^\dagger }{C_*}=\frac{\phi _S}{R_e}(r-1)=(1-R_e^{-1})\frac{\phi _S}{1-\phi _S} \end{aligned}$$

for both the unvaccinated ($*=U$) and vaccinated ($*=V$) subpopulations. This ratio (18) can take any positive value as $\phi _S$ varies from zero (full protection against reinfection) to one (no protection). In particular, if the protection against reinfection is low ($\phi _S \approx 1$), there may be more reinfections than primary infections. Interestingly, this ratio of secondary to primary infections decreases as the vaccination coverage *c* increases. In other words, with more vaccinated individuals, a higher proportion of the total infections are primary infections. The disease has fewer opportunities to reinfect hosts, because a higher vaccination coverage means that the final epidemic size is smaller.

19 $$\begin{aligned} C_U+C_U^\dagger +C_V+C_V^\dagger = \left( 1+(1-R_e^{-1})\frac{\phi _S}{1-\phi _S}\right) (1-c(1-\theta _S))\left( 1+\frac{1}{r} W\left[ -r e^{-r}\right] \right) \end{aligned}$$

are the total infections. In the limit $R_0 \rightarrow \infty$, the right-most factor above tends to 1 (meaning that in the classic SIR model, the full population becomes infected), and so (19) tends to $(1-c(1-\theta _S))/(1-\phi _S)$. This limit can also be derived from first principles: $1-c(1-\theta _S)$ is the initial susceptible size and $1/(1-\phi _S)=1+\phi _S(1+\phi _S(\dots ))$ is the expected number of infections an individual undergoes before becoming immune (this number of infections follows a geometric distribution).

#### Final-Size Results Without Vaccination

Setting $c=0$ gives the final-size results in the absence of vaccination. Assuming $R_0>1$, the cumulative primary infections are

20 $$\begin{aligned} C_U =\left( 1+\frac{1}{r_0} W\left[ -r_0 e^{-r_0}\right] \right) \text { with }r_0 = \frac{R_0-\phi _S}{1-\phi _S}>1. \end{aligned}$$

Similarly, the total reinfections are

21 $$\begin{aligned} C^\dagger _U= \frac{\phi _S}{R_0}(r_0-1)\left( 1+\frac{1}{r_0} W\left[ -r_0 e^{-r_0}\right] \right) \end{aligned}$$

and the total infections are

22 $$\begin{aligned} C_U+C_U^\dagger = \left( 1+(1-R_0^{-1})\frac{\phi _S}{1-\phi _S}\right) \left( 1+\frac{1}{r_0} W\left[ -r_0 e^{-r_0}\right] \right) . \end{aligned}$$

## Model with Leaky Immunity

### Model Description

**Fig. 3:**
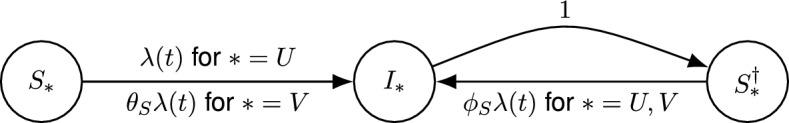
Schematic representation of the SIRI leaky model. The movement of individuals from $S_*$ to $I_*$ differs between the unvaccinated ($*=U$) and vaccinated ($*=V$), as vacinees have lower susceptibility. Flow rates between compartments are given per capita (and with the average infectious period as time unit)

We modify the all-or-none model above to instead account for “leaky” immunity. In this scenario, both infection- and vaccine-induced immunity partially protect against infection, reducing individual host susceptibility but not fully preventing future infections. All hosts with partial immunity can become infected, though at lower rates than those with no immunity. Vaccinees who have never been infected have relative susceptibility $\theta _S \in [0,1]$. Recovered hosts have relative susceptibility $\phi _S \in [0,1]$, regardless of vaccination status: we make this simplifying assumption for tractability. Biologically, we expect $\phi _S \le \theta _S$ so that infection does not increase the susceptibility of vaccinated hosts. With these assumptions, the model (Fig. 2) becomes

23 $$\begin{aligned} \dot{S}_U&= -S_U \lambda (t)&\dot{S}_V&= - \theta _S S_V \lambda (t) \end{aligned}$$

24 $$\begin{aligned} \dot{S}_U^\dagger&= -\phi _S S_U^\dagger \lambda (t) +I_U&\dot{S}_V^\dagger&= - \phi _S S^\dagger _V \lambda (t) +I_V \end{aligned}$$

25 $$\begin{aligned} \dot{I}_U&= (S_U + \phi _S S^\dagger _U) \lambda (t) -I_U&\dot{I}_V&= (\theta _S S_V + \phi _S S^\dagger _V) \lambda (t) -I_V \end{aligned}$$

where $\lambda =R_0(I_U+I_V)$ is the force of infection (as before). Initially, all vaccinees are susceptible to infection. Hence, ${(S_U, S^\dagger _U, I_U)=(1-c, 0, \epsilon _U)}$ and $(S_V, S^\dagger _V, I_V)=(c, 0, \epsilon _V)$ at $t=0$ (again with $0 \le \epsilon _U, \epsilon _V \ll 1$ and $\epsilon _U+\epsilon _V>0$). The initial effective reproduction number is $R_e=R_0(1-c(1-\theta _S))$, as before. Hence, the epidemic outbreak again grows for vaccination coverages *c* below the herd-immunity threshold $c_h=(1-R_0^{-1})/(1-\theta _S)$.

### Final-Size Equation

To derive the final size of the epidemic, the force of infection $\lambda$ is expressed in terms of the number of susceptibles. Substituting for $S^\dagger _U=1-c-S_U-I_U$ and $S^\dagger _V=c-S_V-I_V$ in $\dot{\lambda } = \lambda R_0 [S_U+\phi _S S^\dagger _U+\theta _S S_V+ \phi _S S^\dagger _V] -\lambda$:

26 $$\begin{aligned} \dot{\lambda }&= \lambda R_0 [S_U(1-\phi _S)+S_V(\theta _S - \phi _S )+(\phi _S(1-c)+ \phi _S c)-(\phi _S I_U+ \phi _S I_V)] -\lambda . \end{aligned}$$

We want to express $\dot{\lambda }$ purely in terms of $\lambda$ and $S_U$ to obtain an implicit final-size equation. We eliminate $S_V$ from (26) using

27 $$\begin{aligned} S_V= c \left( \frac{S_U}{1-c} \right) ^{\theta _S} \end{aligned}$$

which follows from (23) and the initial conditions above. The relation (27) is as in Appendix A.2 of Gutierrez and Gog (2023). Substituting (27) in (26):

28 $$\begin{aligned} \dot{\lambda }&=\lambda R_0\left[ S_U(1-\phi _S)+c\left( \frac{S_U}{1-c}\right) ^{\theta _S}(\theta _S -\phi _S)+\phi _S-\phi _S R_0^{-1} \lambda \right] -\lambda . \end{aligned}$$

Using the chain rule and (28),

29 $$\begin{aligned} \frac{d\lambda }{dS_U} = \frac{\dot{\lambda }}{\dot{S_U}} =-R_0 (1-\phi _S)-R_0 c(\theta _S -\phi _S)\frac{S_U^{\theta _S-1}}{(1-c)^{\theta _S}} +S_U^{-1}(1-R_0 \phi _S)+\phi _S \frac{\lambda }{S_U}. \end{aligned}$$

We solve (29) to find $\lambda$ as a function of $S_U$:

30 $$\begin{aligned} \frac{d}{dS_U}[S_U^{-\phi _S} \lambda ] =&S_U^{-\phi _S} \left[ \frac{d\lambda }{dS_U}- \phi _S \frac{\lambda }{S_U} \right] \end{aligned}$$

31 $$\begin{aligned} {=}&S_U^{{-}\phi _S} \left[ {-}R_0 (1-\phi _S){-}R_0 c(\theta _S {-}\phi _S)\frac{S_U^{\theta _S{-}1}}{(1-c)^{\theta _S}} +S_U^{-1}(1-R_0 \phi _S)\right] . \end{aligned}$$

Therefore,

32 $$\begin{aligned}&\lambda - K R_0 S_U^{\phi _S} = -R_0 i_{\{\phi _S \ne 1 \}} S_U-R_0 c \left( \frac{ S_U}{1-c}\right) ^{\theta _S} i_{\{\phi _S \ne \theta _S \}}-(\phi _S^{-1}-R_0) i_{\{\phi _S \ne 0 \}} \nonumber \\&\qquad -i_{\{\phi _S = 0 \}} \log {\frac{1-c}{S_U}}. \end{aligned}$$

where $i_{\{Y\}}$ is the indicator function (equal to one if the statement *Y* is true, and zero otherwise) and *K* is a constant of integration, set by the initial conditions $\lambda (0) = 0$ and $S_U(0)=1-c$:

33 $$\begin{aligned} K =(1-c)^{-\phi _S}\left[ (1-c)i_{\{\phi _S \ne 1 \}} +c i_{\{\phi _S \ne \theta _S \}}+\left( R_0^{-1} \phi _S^{-1}-1\right) i_{\{\phi _S \ne 0 \}}\right] . \end{aligned}$$

Therefore,

34 $$\begin{aligned} \lambda = R_0&\left[ \left( \frac{ S_U}{1-c}\right) ^{\phi _S} \left[ (1-c)i_{\{\phi _S \ne 1 \}} +c i_{\{\phi _S \ne \theta _S \}}+\left( R_0^{-1} \phi _S^{-1}-1\right) i_{\{\phi _S \ne 0 \}}\right] - i_{\{\phi _S \ne 1 \}} S_U \right. \nonumber \\&\left. -c \left( \frac{ S_U}{1-c}\right) ^{\theta _S} i_{\{\phi _S \ne \theta _S \}}-(R_0^{-1} \phi _S^{-1}-1)i_{\{\phi _S \ne 0 \}} \right] -i_{\{\phi _S = 0 \}} \log {\frac{1-c}{S_U}} \end{aligned}$$

35 $$\begin{aligned} = R_0&\left[ \left( \frac{ S_U}{1-c}\right) ^{\phi _S}- S_U -c \left( \frac{ S_U}{1-c}\right) ^{\theta _S} +\left( \left( \frac{ S_U}{1-c}\right) ^{\phi _S}-1 \right) \left( \frac{1}{R_0 \phi _S}-1\right) i_{\{\phi _S \ne 0 \}}\right] \nonumber \\&-i_{\{\phi _S = 0 \}} \log {\frac{1-c}{S_U}}. \end{aligned}$$

Setting $\lim _{t \rightarrow \infty } \lambda (t) = 0$ in (35) gives an implicit equation for the final size $S_U^{\infty } = \lim _{t \rightarrow \infty } S_U (t) <1-c$. If $\phi _S=0$, reinfections are impossible. Thus, with $\phi _S=0$ we would obtain the final-size equation (43) of Gutierrez and Gog (2023), here with $\theta _I=1$. For $\phi _S>0$, the final-size equation is

36 $$\begin{aligned} S_U^{\infty } =(R_0 \phi _S)^{-1}\left( \frac{S_U^{\infty }}{1-c}\right) ^{\phi _S}-c\left( \frac{S_U^{\infty }}{1-c}\right) ^{\theta _S}-\left[ (R_0 \phi _S)^{-1}-1\right] . \end{aligned}$$

We show below that (36) has a solution $S_U^\infty <1-c$ for $R_0 \phi _S <1$. If $R_0 \phi _S >1$, the epidemic reaches a stable endemic equilibrium—so $R_0 =1/\phi _S$ is a “reinfection threshold” (Gomes et al. 2005, 2004)—in which all hosts have been infected before ($S_U=0=S_V$, analogous to the endemic equilibrium of the standard SIS model (Keeling and Rohani 2007) for $S_U^{\dagger }, S_V^{\dagger }$ and $I_U, I_V$). In contrast, the all-or-none model does not have an endemic equilibrium (the last paragraph of Section 5 discusses this important difference). Here we are interested in a transient epidemic with both primary infections and reinfections, so henceforth we assume $R_0 \phi _S<1$.

#### Existence and Uniqueness of the Solution to the Final-Size Equation

The initial condition $S_U(0)=1-c$ for the susceptible size before the outbreak solves (36). We now prove that (36) also has a solution $S_U^{\infty } \in (0, 1-c)$, provided that $R_0 < 1/\phi _S$ (see previous paragraph) and that the vaccination coverage is below the threshold for herd immunity ($c< c_h=(1-R_0^{-1})/(1-\theta _S)$). We define $F(x)=(R_0 \phi _S)^{-1} \left( {x}/({1-c})\right) ^{\phi _S}-c\left( {x}/({1-c})\right) ^{\theta _S}-[(R_0 \phi _S)^{-1}-1]$ so that $x=S_U^{\infty }$ is a fixed point of $F(x)=x$. We also define $g(x)=F(x)-x$, so that $g(1-c)=0$ and

37 $$\begin{aligned} \frac{dg}{dx}(1-c)=g'(1-c)=\frac{R_0^{-1}-c\theta _S}{1-c}-1. \end{aligned}$$

Using $c<c_h=(1-R_0^{-1})/(1-\theta _S)$, it follows that $g'(1-c)<0$. Since $g(1-c)=0$, we can pick $L>0$ such that $g(1-c-\eta )>0$ for all $\eta \in (0,L]$. Moreover, $g(0)=-[(R_0 \phi _S)^{-1}-1]<0$. By the intermediate value theorem (applied to *g*(*x*), which is a continuous function of *x*), there exists a solution $S_U^{\infty } \in (0, 1-{c}-L)$ to $g(S_U^{\infty })=0 \iff F(S_U^{\infty })=S_U^{\infty }$, the final-size equation (36). To prove the uniqueness of $S_U^{\infty }$, we use the mean value theorem. If $x_1=F(x_1)< x_2=F(x_2)$, then there exists $x_* \in (x_1, x_2)$ such that $F'(x_*)=(F(x_1)-F(x_2))/(x_1-x_2)=1$. For $x_*>0$, $F'(x_*)=1$ is equivalent to

38 $$\begin{aligned} (1-c)\left( \frac{x_*}{1-c}\right) ^{1-\phi _S}+c\theta _S \left( \frac{x_*}{1-c}\right) ^{\theta _S-\phi _S}=R_0^{-1}. \end{aligned}$$

Since $\theta _S \ge \theta _S^{\dagger }=\phi _S$, both exponents in (38) are non-negative. Therefore, the left-hand side of (38) monotonically increases with increasing $x_*$: from zero at $x_*=0$ (or if $\theta _S=\phi _S$, from $c\phi _S \le \phi _S < 1/R_0$), to $(1-c)+c\theta _S>R_0^{-1}$ at $x_*=1-c$. Hence, (38) has only one solution $x_* \in (0,1-c)$. Thus, *F*(*x*) has at most two fixed points $x_1,x_2 \in [0,1-c]$. Since $1-c$ is a fixed point, we must have $x_2=1-c$, so $x_1=S_U^{\infty }=F(S_U^{\infty })<1-c$ is unique.

### Final-Size Results

For $R_0 \phi _S < 1$, the final number of unvaccinated individuals who never become infected is the solution $S_U^{\infty }<1-c$ of (36). The cumulative primary infections are $C_U=1-c-S_U^{\infty }$ (in the unvaccinated) and $C_V=c-S_V^{\infty }$ (in the vaccinated), with $S_V^\infty =c\left( S_U^{\infty }/(1-c) \right) ^{\theta _S}$ following from (27). Therefore, the total primary infections are

39 $$\begin{aligned} C_U+C_V =((1-c)-S_U^{\infty }) +c\left[ 1-\left( \frac{S_U^{\infty }}{1-c} \right) ^{\theta _S} \right] = \frac{1}{R_0 \phi _S}\left[ 1-\left( \frac{S_U^{\infty }}{1-c} \right) ^{\phi _S}\right] . \end{aligned}$$

The total infections are

40 $$\begin{aligned} C_U+C_U^\dagger +C_V+C_V^\dagger&= \int _0^{\infty } (S_U + \phi _S S^\dagger _U+\theta _S S_V+\phi _S S^\dagger _V) \lambda dt \nonumber \\&= \int _0^{\infty } R_0^{-1} \lambda dt = \frac{1}{R_0} \log {\frac{1-c}{S_U^{\infty }}}, \end{aligned}$$

where we have used the expressions for $\dot{\lambda }$ (given above by (26)) and $\dot{S}_U$. Thus, the cumulative infections in hosts with prior immunity are

41 $$\begin{aligned} C_U^\dagger +C_V+C^\dagger _V =\frac{1}{R_0} \log {\frac{1-c}{S_U^{\infty }}}-C_U=\frac{1}{R_0} \log {\frac{1-c}{S_U^{\infty }}}-((1-c)-S_U^{\infty }). \end{aligned}$$

and—subtracting (39) from (40)—the cumulative reinfections are

42 $$\begin{aligned} C_U^\dagger +C_V^\dagger = \frac{1}{R_0} \log {\frac{1-c}{S_U^{\infty }}} -\frac{1}{R_0 \phi _S}\left[ 1-\left( \frac{S_U^{\infty }}{1-c} \right) ^{\phi _S} \right] . \end{aligned}$$

We have not been able to find expressions for $C_U^\dagger$ and $C_V^\dagger$ alone.

#### Final-Size Results Without Vaccination

Setting $c=0$ simplifies the final-size equation (36), which is valid for $R_0 \phi _S<1$, to

43 $$\begin{aligned} S_U^{\infty } =(R_0 \phi _S)^{-1}\left( {S_U^{\infty }}\right) ^{\phi _S}-\left[ (R_0 \phi _S)^{-1}-1\right] . \end{aligned}$$

Thus, the cumulative primary infections are $C_U=1-S_U^{\infty }$, where $S_U^{\infty }$ is the solution $S_U^{\infty }<1$ of (43). Similarly, the total infections are

44 $$\begin{aligned} C_U+C_U^\dagger = \frac{1}{R_0} \log {\frac{1}{S_U^{\infty }}} \end{aligned}$$

and the total reinfections are

45 $$\begin{aligned} C_U^\dagger =\frac{1}{R_0} \log {\frac{1}{S_U^{\infty }}}-(1-S_U^{\infty }). \end{aligned}$$

## Discussion

**Fig. 4:**
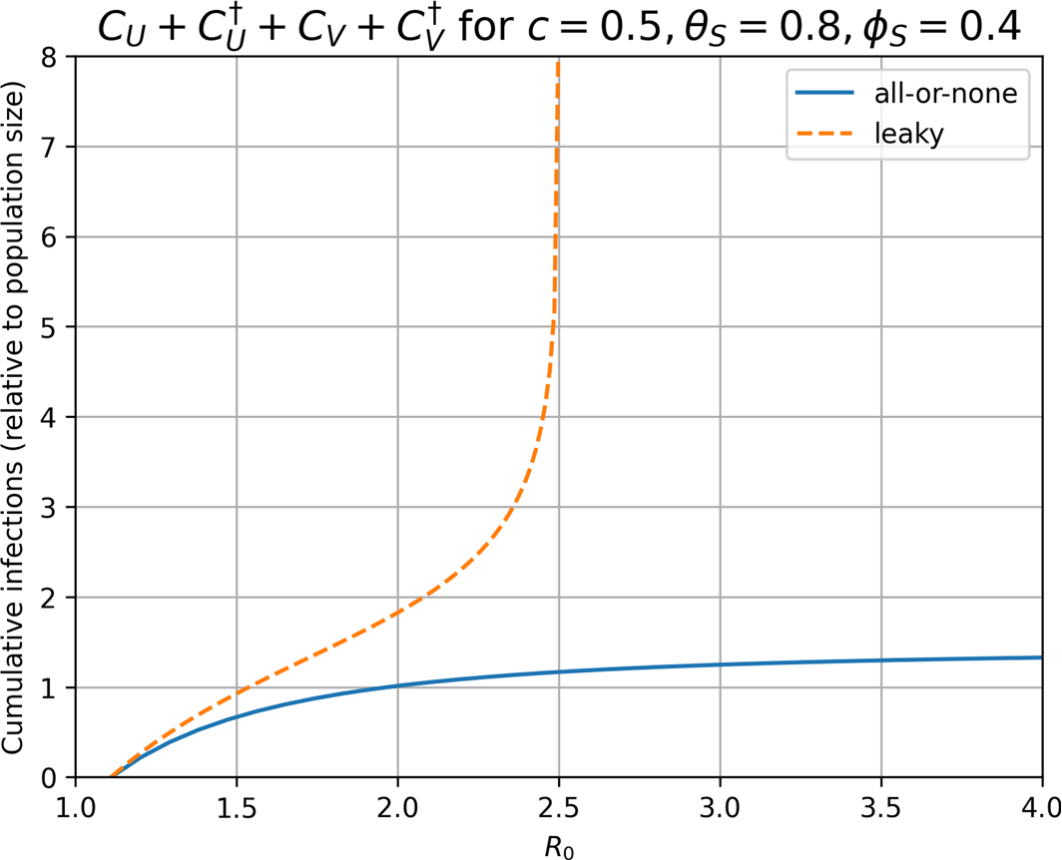
Cumulative number of infections for the leaky and all-or-none models, for fixed vaccination coverage $c=0.5$, and susceptibilities $\theta _S=0.8, \phi _S=0.4$. We show values of $R_0$ above $1/(1-c(1-\theta _S))=10/9$ so that the initial effective reproduction number is greater than one ($R_e>1$). With leaky immunity, the final size approaches infinity as $R_0$ approaches the reinfection threshold (here $\phi _S^{-1}=2.5)$. With all-or-none immunity, as $R_0 \rightarrow \infty$, the final size (19) approaches a constant $(1-c(1-\theta _S))/(1-\phi _S)=3/2$, as explained in Section 3.3

In Section 3, we have studied an epidemic model including reinfections, in which immunity (from both infection and vaccination) is “all-or-none” (Halloran et al. 1999). The all-or-none immunity assumption facilitates the mathematical analysis, because there are inherently still only two immunity statuses in the model (fully immune or fully susceptible). While this form of immunity has been used to consider partial cross-immunity between distinct antigenic strains (Gog and Grenfell 2002; Kucharski et al. 2016), we are not aware of other models purely of reinfection that consider this all-or-none form.

The assumption that immunity against reinfection is “leaky” has been considered in other models before (Moreira and Yuquan 1997; Gökaydin et al. 2007; Gomes et al. 2005, 2004). Since we include vaccination, the model in Sect. 4 is most similar to the model for “partial immune protection and vaccination” of Gomes et al. (2004), but there are two key differences. First, because we do not model the birth-and-death dynamics of the population, we do not consider vaccination at birth. Instead, we capture the vaccination coverage of the population through the initial conditions of an epidemic (as in Sect. 3). Second, we assume the same susceptibility to reinfection for all hosts, regardless of vaccination status. We assume that this susceptibility to reinfection is not higher than the susceptibility of vaccinees who have never been infected. In contrast, Gomes et al. (2004) assumes that vaccinees always have the same susceptibility, regardless of whether they have been infected before or not, and that this susceptibility is lower than that of recovered uninfected individuals. Thus Gomes et al. (2004) considers vaccines that protect against infections at least as much as infection-induced immunity, while Sect. 4 considers vaccines that protect no more than infection-induced immunity.

As in Gomes et al. (2004), we find a “reinfection threshold” (RT) for $R_0$, separating two qualitatively different epidemiological scenarios. However, the RT in Sect. 4 is always determined by the relative susceptibility to reinfection $\phi _S$: unlike the RT of Gomes et al. (2004), the RT here does not depend on the relative susceptibility of vaccinees. Moreover, in Gomes et al. (2004), the RT only separates two quantitative regimes for the same endemic equilibrium, so it is not a bifurcation point of the dynamical system (Breban and Blower 2005). In contrast, the RT here is a bifurcation point as it separates a transient epidemic wave ($R_0<1/\phi _S$) from an endemic equilibrium ($R_0>1/\phi _S$). The endemic state occurs if the infection-induced protection against reinfection is low relative to the transmission rate ($\phi _S >R_0^{-1}$). In this endemic equilibrium state, all hosts have previously been infected. Since this endemic state does not arise in the all-or-none model, this work highlights the importance of the mode in which immunity acts (leaky or all-or-none, although reality is likely somewhere between these two extremes (Gomes et al. 2014)).

In both the all-or-none and leaky, a recovered host has the same relative probability ($\theta _S$) of becoming infected the first time it is re-challenged with infection. However, in subsequent challenges, the models differ. On the one hand, a population with leaky immunity always has the potential to be infected further under sufficient force of infection (each individual host, upon sufficient exposure to the pathogen, could be infected infinitely many times). On the other hand, the all-or-none form means a limit to the capacity for reinfection, with eventually all hosts fully immune under a sustained force of infection. This dynamic difference is analogous to the effects of partial cross-immunity between pathogen strains: sustained oscillations are possible with the leaky form under certain parameters, but never with the all-or-none form (Dawes and Gog 2002). The parallel in this work is that the epidemic is always transient under reinfection with all-or-none immunity but can be sustained for leaky immunity. Similarly, paramaters leading to a transient epidemic under leaky immunity produce a smaller final epidemic size under all-or-none immunity (Figure 3).

Both epidemic models above group together all infectious hosts with the same vaccination status, regardless of how many times (if any) they have been infected before. This assumption is needed to limit the number of model compartments. Some of the implicit assumptions are that there are no differences between (i) host infectiousness during primary infections and reinfections, (ii) the infectious period during primary infections and reinfections, or (iii) the immunity induced by primary infections and reinfections. Point (i) means that we ignore both the infection-induced and vaccine-induced reduction in host infectiousness. Point (ii) means that we assume that infection-induced immunity does not shorten future infections, matching the analogous assumption for vaccine-induced immunity. This assumption is likely simplifying reality: hosts clear SARS-CoV-2 faster during reinfections (Kissler et al. 2023). While the exact implications for infectivity are unclear, prior immunity probably affects the duration of infectivity. Point (iii) implies that we ignore immune imprinting and, more generally, the accumulation of immunity from multiple infections. Hence, at the beginning of each reinfection, the immune status of each host is the same, irrespective of whether it is the first or a later reinfection.

Moreover, we do not account for hybrid immunity (Crotty 2021). In the model with leaky immunity, the susceptibility to reinfection is independent of the host’s vaccination status. In other words, the vaccine-induced immunity does not protect recovered hosts, only the infection-induced immunity does. For the model with all-or-none immunity, there is a similar underlying assumption. If a host is infected, the probability of acquiring (full) protection against reinfection does not depend on their vaccination status. This assumption is also needed to obtain an analytical final-size expression (if vaccinees had a lower probability of becoming susceptible to reinfection, (5) would not hold). Neglecting hybrid immunity means that the results here underestimate the effects of vaccination in reducing the number of infections.

Finally, both models allow reinfections immediately after recovery from infection. There is some evidence suggesting that hosts may experience two infections in short succession with the same strain of Influenza A (Memoli et al. 2020) and SARS-CoV-2 (Tillett et al. 2021). However, most reinfections likely occur due to a combination of antigenic escape (de Jong et al. 2000; Yewdell 2021) and waning immunity (Bull et al. 2025). We do not model waning immunity explicitly because we intended to focus on the dynamics of reinfection. Similarly, sequential reinfections (i.e., without waning of immunity) of the same strain are also possible in other models studying the evolution of Influenza A (Gökaydin et al. 2007; Gordo et al. 2009) and SARS-CoV-2 (Saad-Roy et al. 2024; Rella et al. 2024).

In this paper we have obtained final-size solutions of SIRI-type epidemic models with partial infection-induced immunity, which leads to reinfections. These models also include partial vaccine-induced protection against (primary) infections for a fraction of the population that has been vaccinated before the outbreak. Assuming all-or-none immunity, we obtain analytic final-size solutions in terms of the Lambert W function. These solutions have a similar mathematical structure to the final-size solution of the standard SIR model, but account for partial infection-induced immunity. Assuming instead leaky immunity, we find implicit solutions for the final epidemic size. These solutions (for a transient epidemic) are only valid if the susceptibility to reinfection is relatively low. If instead the susceptibility to reinfection is above a reinfection threshold, the system reaches an endemic state (similar to an SIS model). These results could help predict the epidemiological impact of disease that only induce partial protection in recovered hosts. For example, our final-size solutions quantify the reduction in the cumulative infections that would occur if a fraction of the population was vaccinated (prior to the outbreak). This reduction varies depending on the intrinsic transmissibility of the pathogen and the relative susceptibilities of vaccinees and recovered hosts.

## Data Availability

There are no supporting data.
